# The adipokine C1q TNF related protein 3 (CTRP3) is elevated in the breast milk of obese mothers

**DOI:** 10.7717/peerj.4472

**Published:** 2018-03-05

**Authors:** Megan R. Kwon, Eileen Cress, W. Andrew Clark, Arsham Alamian, Yongke Lu, Jonathan M. Peterson

**Affiliations:** 1 Department of Allied Health Sciences, College of Clinical and Rehabilitative Health Sciences, East Tennessee State University, Johnson City, TN, USA; 2 James H. Quillen VA Medical Center, Mountain Home, TN, USA; 3 Department of Biostatistics and Epidemiology, College of Public Health, East Tennessee State University, Johnson City, TN, USA; 4 Department of Health Sciences, College of Public Health, East Tennessee State University, Johnson City, TN, USA

**Keywords:** CTRP3, Dipokine, Breast milk, Obesity

## Abstract

**Background:**

C1q TNF related protein 3 (CTRP3) is a relatively novel hormonal factor primarily derived from adipose tissue and has anti-diabetic properties. To determine if CTRP3 could play a role in early childhood development, the purpose of this study was to establish the presence of CTRP3 in breast milk (BM) and to determine whether CTRP3 levels were correlated with pregravid obesity status of the mother.

**Methods:**

Breast milk was collected from breast-feeding mothers who had a pregravid body mass index (BMI) classification of normal weight (BMI 18–25 kg/m^2^, *n* = 23) or obese (BMI > 30 kg/m^2^, *n* = 14). Immunoprecipitation followed by immunoblot analysis confirmed the presence of CTRP3 in BM. The concentration of CTRP3 in BM samples was determined by ELISA. Additional bioactive components were also measured by commercially available assays: ghrelin, insulin, leptin, adiponectin, interleukin-6 (IL-6), tumor necrosis factor-alpha (TNF-α), and glucose. Bioactive components in normal weight and obese mothers were compared using unpaired *t*-test (parametric) and Mann–Whitney *U*-test (non-parametric), as appropriate.

**Results:**

The primary findings of this study are that the adipokine CTRP3 is present in BM and CTRP3 levels are increased with pregravid obesity. Additionally, this study independently confirmed previous work that BM from obese mothers has a higher concentration of insulin and leptin. Further, no differences were observed in BM between obese and normal weight mothers in ghrelin, adiponectin, IL-6, TNF-α, or glucose levels.

**Conclusion:**

This study identified a novel factor in BM, CTRP3, and showed that BM CTRP3 levels higher in obese mothers. Because of the purported insulin sensitizing effect of CTRP3, it is possible that the elevated levels of CTRP3 in the BM of obese mothers may offset negative effects of elevated leptin and insulin levels in the BM of obese mothers. Future studies will need to be conducted to determine the relevance of CTRP3 in BM and to examine the presence of other adipose tissue-derived hormonal factors.

## Introduction

Childhood obesity is a growing epidemic in the Unites States (U.S.), with prevalence increasing from ∼7% in 1980 to 17.7% in 2012 (National Center for Health Statistics (U.S.), 2015; Ogden et al., 2014; Troiano & Flegal, 1998). Further, obese children are at a significantly higher risk for developing many types of cancers, cardiovascular disease, diabetes, and metabolic syndrome (MetS) (Ekelund et al., 2009; Friend, Craig & Turner, 2013; Maligie et al., 2012). Therefore, understanding factors that contribute to childhood obesity are of great importance. Epidemiological evidence suggests breastfeeding protects against obesity and metabolic disorders, however the cause(s) of these associations are unknown (Gillman, 2002; Global BMI Mortality Collaboration et al., 2016; Harder et al., 2005; Horta, Loret de Mola & Victora, 2015; Kalliomaki et al., 2008; Lemas et al., 2016; Taveras et al., 2011; Toschke et al., 2002; Yan et al., 2014; Young et al., 2017; Zalewski et al., 2017). Further, the breastfeeding-derived defense against infant obesity and metabolic disorders is not universal across all lactating mothers (Woo & Martin, 2015). BM is a complex solution that contains the nutrients needed to sustain growth and development of a newborn. In addition, BM contains a variety of non-nutritive bioactive components (e.g., hormones, cytokines, etc.) that can influence metabolic function, which may explain the beneficial effects of breastfeeding (Fields, Schneider & Pavela, 2016; Garcia-Mantrana & Collado, 2016; Lemas et al., 2016; Socha et al., 2016).

On the other hand, animal studies have indicated the potential for BM to promote the transfer of obesity from mother to offspring (Del Prado, Delgado & Villalpando, 1997; Mackay et al., 2013; Matsuno et al., 1999; Oosting et al., 2015; Palou et al., 2010; Sun et al., 2012; Tsuduki et al., 2013). The influence of factors within the BM, which could contribute to the maternal transfer of obesity, have rarely been studied. One potential development is the presence of adipose tissue-derived hormonal factors in BM, such as adiponectin and leptin (Casabiell et al., 1997; Çatli, Olgaç Dündar & Dündar, 2014; Fields, Schneider & Pavela, 2016; Garcia-Mantrana & Collado, 2016; Lemas et al., 2016; Sanchez et al., 2005; Savino et al., 2002; Socha et al., 2016). Adipose tissue is a dynamic tissue that secretes many bioactive molecules, collectively termed adipokines, and many adipokines have been identified in BM (Casabiell et al., 1997; Çatli, Olgaç Dündar & Dündar, 2014; Fields & Demerath, 2012; Fields, Schneider & Pavela, 2016; Lemas et al., 2016; Young et al., 2017). It is well documented that dysregulation of adipokines can lead to inflammation, insulin resistance, atherosclerosis, hypertension, hyperphagia (overeating), and the development of MetS (Pardo et al., 2012; Rutkowski, Stern & Scherer, 2015). Recently published studies have reported changes to a number of hormones, specifically leptin and insulin, in BM from obese compared with normal weight mothers (Kugananthan et al., 2017; Lemas et al., 2016; Ley et al., 2012). Others have identified associations with adipokine levels in BM and infant growth (Çatli, Olgaç Dündar & Dündar, 2014; Chan et al., 2017; Fields, Schneider & Pavela, 2016; Khodabakhshi et al., 2017; Savino et al., 2002). Due to the permeability of the digestive tract in early infancy non-nutritive bioactive factors, such as adipokines, can be transferred to the infant’s circulation (Sanchez et al., 2005; Shehadeh, Sukhotnik & Shamir, 2006), where they are positioned to influence infant physiology. The presence of these adipokines in BM suggests that they contribute to the regulation of development and energy balance in early infancy. Further, deregulation of these factors could contribute to the development of metabolic disorders in childhood and adulthood.

Recently a family of novel secreted humoral factors, C1q TNF related proteins (abbreviated CTRP1-15) have been identified. The initial characterization of these adipose tissue-derived CTRP factors established wide-ranging effects on stimulating metabolism and preventing inflammation (Peterson et al., 2012; Peterson, Wei & Wong, 2010; Wong et al., 2004; Yoo et al., 2013). In particular, C1q TNF related protein 3 (CTRP3) is a novel exciting and unique adipokine which has demonstrated a number of anti-diabetic properties, specifically in regard to lipid metabolism and cardiovascular function (Li et al., 2016; Petersen et al., 2016; Peterson et al., 2013; Peterson, Wei & Wong, 2010; Yoo et al., 2013). The purpose of this manuscript is two-fold: first, is to establish the presence of CTRP3 in breast milk (BM); second, is to determine whether the concentration of CTRP3 is different in BM from normal weight compared with obese mothers.

## Methods

### Study design and subjects

This descriptive study derives from BM collected from women who had a self-reported normal weight pregravid body mass index (BMI) in kg/m^2^ of 18–25 (normal weight) or ≥30 (obese), 18–45 years of age, and between two and 14 weeks of lactation. Subjects were recruited from the Breastfeeding Advocacy Benefits Everyone (BABE) Breastfeeding Coalition of Northeast Tennessee (http://breastfeedingsupportnet.com/providers/providers_about.asp). Self-reported pregravid height and weight have been reported to be highly correlated with direct physical measurements (Shin et al., 2014), and for the purposes of this study were sufficient to categorize participants as either normal weight or obese. Signed written informed consent was obtained from all participants and all study procedures were approved by East Tennessee State University’s Institutional Review Board (IRB No 0915.8s, 2016). Exclusion criteria included BMI < 18 kg/m^2^ or 26–30 kg/m^2^, diagnoses with type 1 diabetes, non-breastfeeding, or other significant health issue requiring medical treatment.

### Sample collection and preparation

Subjects were screened to confirm eligibility and instructed to pump at home immediately prior to their regularly scheduled BABE group meeting. Subjects transported the samples on ice where they were collected and immediately transported (on ice) to the lab. At the lab they were vortexed and skim milk was prepared by centrifugation (20,000*g*, 15 min, 4 °C). Skim milk was stored at −80 °C until further analysis. All samples were collected prior to start of the meeting and reported to have been produced within 2 h of collection by investigators.

### CTRP3 immunoprecipitation

Co-immunoprecipitation (Co-IP) procedures were performed at 4 °C unless otherwise indicated, using a Dynabeads® Protein A magnetic beads, according to manufacturer’s instructions (Cat#10016D; Thermo Fisher Scientific, Waltham, MA, USA). Briefly, 50 μl of Dynabeads®-Ab complex bead slurry were prepared with 5 μg rabbit polyclonal anti-CTRP3 (GW Wong Lab; Johns Hopkins University Cat# WongLab_anti-gM3 Lot# RRID:AB_2572292) (Peterson, Wei & Wong, 2010), 5 μg rabbit polyclonal anti-CTRP1 (GW Wong Lab; Johns Hopkins University Cat# anti-gCTRP1, RRID:AB_2716247) (Wong et al., 2008), 10 μl normal goat serum (Cat# 10000C; Thermo Fisher Scientific, Waltham, MA, USA, RRID:AB_2532979), or phosphate-buffered saline (PBS) alone. The antibody-bead slurry was rotated for 15 min at room temperature and then separated from the solution using the magnetic stand. The beads were then washed with PBS+T (pH 7.4 with 0.02% Tween®-20), before the addition of 50 μl BM (diluted 1:10 in PBS, 500 μl final volume). The solution was then incubated for 4 h at 4 °C with rotation to allow CTRP3 to bind to the Dynabeads®-Ab complex. The tubes were returned to the magnet and the supernatant (unbound protein solution) was collected and denatured at 70 °C for 10 min after the addition of an equal amount of 2× SDS loading buffer (4% SDS, 10% 2-mercaptoethanol, 5 mM DTT, 20% glycerol, 0.004% bromophenal blue, 0.125M Tris–HCl, pH 6.8). The Dynabeads®-antibody-CTRP3 complex were then washed three times in supplied wash buffer. Next, CTRP3 was removed from the beads with the addition of 20 μl of the vendor supplied elution buffer and 20 μl 2× SDS loading buffer and heating at 70 °C for 10 min. The tube was placed on the magnetic rack and the supernatant was collected as the immunoprecipitate solution.

### Immunoblot analysis

The unbound protein solution and immunoprecipitate solution were loaded unto an SDS-polyacrylamide gel and proteins were separated by electrophoresis, according to manufactures directions (Cat#456-1046; Bio-Rad Laboratories, Inc., Hercules, CA, USA) with the addition of a protein ladder (Cat#161-0374; Bio-Rad Laboratories, Inc., Hercules, CA, USA). After gel electrophoresis proteins were transferred to a nitrocellous membrane according to standard procedures (Cat# 1620115; Bio-Rad Laboratories, Inc., Hercules, CA, USA). To reduce non-specific protein interactions the membrane was then blocked in 4% non-fat milk for 1 h at room temperature, and then probed with verified goat anti-CTRP3 primary antibody (Cat# AF2436; R and D Systems, Minneapolis, MN, USA, RRID:AB_2067713) followed by HRP-conjugated rabbit anti-goat secondary antibody (Cat# 31402; Thermo Fisher Scientific, Waltham, MA, USA, RRID: AB_228395). Chemiluminescent signals were detected (chemiluminescent HRP substrate, Cat# WBKLS0100; Millipore, Burlington, MA, USA) and quantified using the Alphaview software and FluorChem M Western Imaging System (Proteinsimple, San Jose, CA, USA).

### Analysis of bioactive compounds in BM

Ghrelin, insulin, leptin, adiponectin, interleukin-6 (IL-6), and tumor necrosis factor-alpha (TNF-α) were measured with Bio-Plex® Multiplex Immunoassay System (Bio-Rad Bio-Plex Cat# 171A7001M, 171A7002M, 171B5006M, and #171B5026M; Bio-Rad, Hercules, CA, USA). CTRP3 levels were determined by commercially available human CTRP3 ELISA kit (Cat# SK00082-07; Aviscera Biosciences, Santa Clara, CA, USA) according to manufacturer’s protocol. Glucose levels were measured by glucose assay (Cat# CBA086; Millipore, Burlington, MA, USA). All assays were performed on samples diluted 1:2 in provided kit specific assay buffer, and performed otherwise according to manufactures directions. The intra- and inter-assay coefficients of variation for all assays were ≤10% and ≤6%, respectively

### Statistical analysis

Descriptive statistics were calculated for age, height, pregravid body weight, parity, postpartum day of lactation, and for all measured BM variables. A D’Agostino and Pearson omnibus normality test was performed on all variables and the data for ghrelin, insulin, leptin, IL-6, TNF-α, and CTRP3 were skewed. All remaining variables were normally distributed: Age, height, weight, BMI, glucose, and adiponectin. Therefore, differences in skewed data were compared by Mann–Whitney *U*-test and normally distributed data were compared by unpaired *t*-test. Correlations between measured variables and CTRP3 were analyzed by Spearman nonparametric correlation test. All data are reported as means and standard error. All statistical analysis was performed by Graphpad Prism 6.

## Results

### Subject characteristics

Thirty-seven women with a pregravid BMI (mean: 27.4 ± 7.0 kg/m^2^; range of 19.6–45.5 kg/m^2^) enrolled into the study. The ethnic distribution of the sample was Caucasian (97.2%) and African–American (2.7%). Subject descriptive statistics are presented in Table 1.

**Table 1:** Subject characteristics.

	Normal weight (*n* = 23)	Obese (*n* = 14)
Maternal age (year)	29.0 ± 1.0	29.6 ± 1.1
Parity	2.0 ± 0.2	2.1 ± 0.2
Postpartum day of lactation	59.7 ± 6.0	52.6 ± 9.4
Height (cm)	165.9 ± 1.22	164.5 ± 1.6
Pregravid weight (kg)	61.2 ± 1.1	98.0 ± 2.8*
Pregravid BMI (kg/m^2^)	22.02 ± 0.4	36.3 ± 1.1*

**Note:**

* Denotes significant difference between groups (*p* < 0.05).

### Verification of CTRP3 in human BM

**Figure 1:**
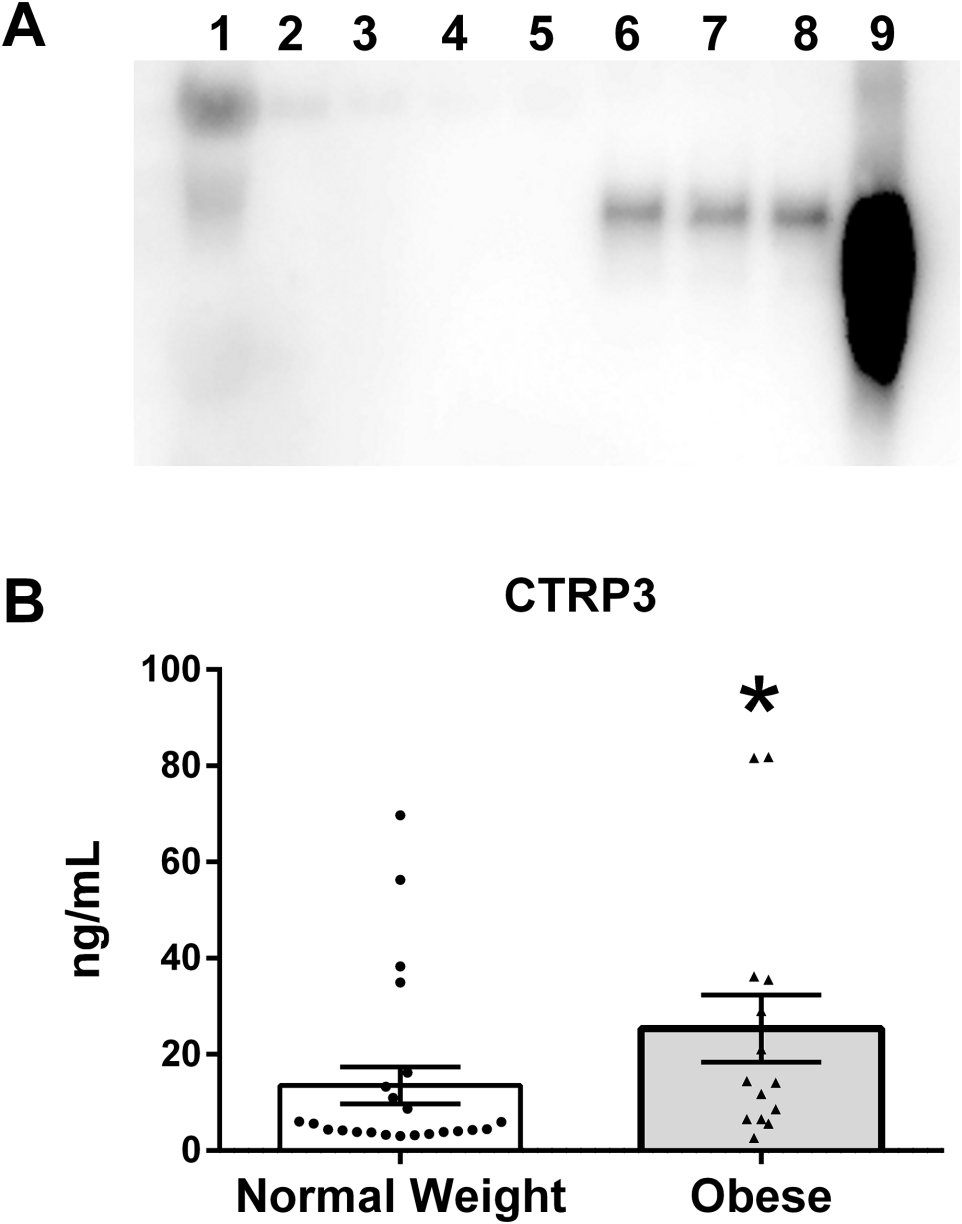
CTRP3 in human breast milk (BM). (A) Immunoblot analysis of whole BM (lane 1), unbound protein solution samples (lanes 2–5) and immunoprecipitate samples (lanes 6–9). BM samples were immunoprecipitated with vehicle only (lanes 2 and 6), normal serum (lanes 3 and 7), anti-CTRP1 (lanes 4 and 8), or anti-CTRP3 antibody (lanes 5 and 9). (B) BM CTRP3 concentration in normal weight compared with obese mothers (*p < 0.05 vs. normal weight group). Values are mean ± SEM, and dots, and triangles represent individual data points from individual participants.

To verify that CTRP3 was present in human BM an immunoprecipitation reaction with rabbit anti-CTRP3 antibody was performed, followed by an immunoblot using a second (goat polyclonal) CTRP3 antibody. This approach was able to detect a specific enrichment of CTRP3, thus demonstrating that CTRP3 is present in human BM (Fig. 1A). Analysis of the BM samples showed a large variation in the concentration of CTRP3, which did not follow a normal distribution: mean 18.0 ng/mL, standard deviation 22.1 ng/mL, range 2.6–81.8 ng/mL, median 6.5 ng/mL, 25% percentile 4.1 ng/mL, and 75% percentile 25.0 ng/mL. A Mann–Whitney *U*-test showed a significant difference in the means of BM CTRP3 levels between obese and normal weight mothers (Fig. 1B).

### Differences in BM composition

**Figure 2:**
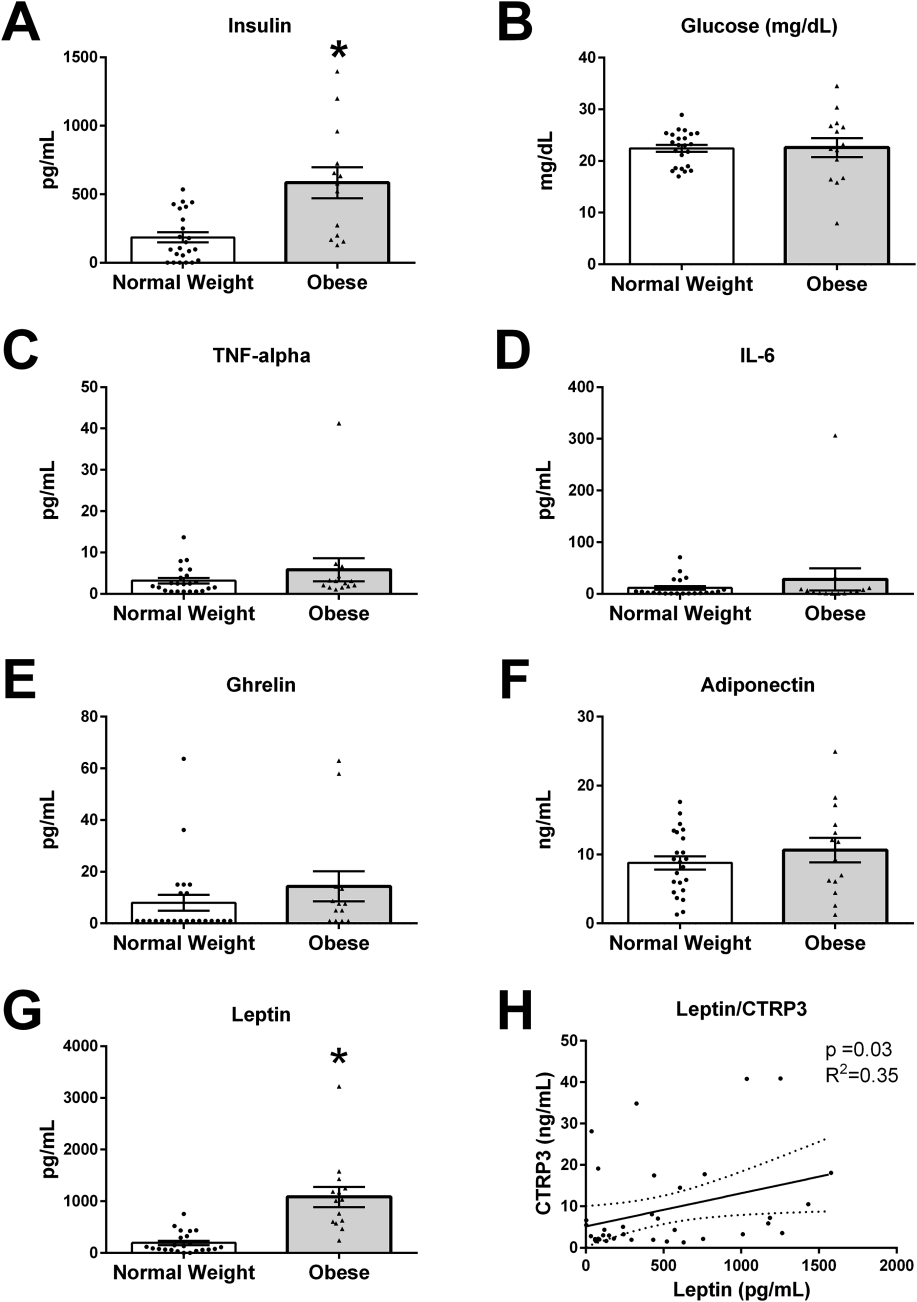
Humoral factors in breast milk (BM). The BM samples from 23 pregravid normal weight (BMI 18–25 kg/m^2^) and 14 obese (BMI > 30 kg/m^2^) mothers between 2 and 14 weeks of lactation were measured for (A) insulin, (B) glucose, (C) TNF-α, (D), IL-6, (E) ghrelin, (F) adiponectin, and (G) leptin (*p < 0.05 vs. normal weight group). Values are mean ± SEM, and dots, and triangles represent individual data points from each participant. (H) Associations between BM and CTRP3 and leptin protein concentration. Solid line (*p* = 0.03, *R*^2^ = 0.35, *n* = 37).

The values for all measured analytes in BM samples are presented in Fig. 2, with values for each participant reported in supplemental data (Table S1). BM concentration of glucose (*p* = 0.19), TNF-α (*p* = 0.23), IL-6 (*p* = 0.74), ghrelin (*p* = 0.13), and adiponectin (*p* = 0.66) were not different between normal weight and obese mothers. However, insulin levels were three times higher in BM from obese mothers (normal weight 186.1 ± 37.0 vs obese 564.3 ± 107.3 pg/mL, *p* < 0.01). Further, leptin levels were ∼5-times higher in BM from obese mothers than normal weight (normal weight 193.8 ± 41.5 vs obese 1,083 ± 194.3 pg/mL, *p* < 0.0001).

### Correlations of BM CTRP3 concentrations and biological variables

In order to examine biological mechanisms for CTRP3, correlations of all measured variables were determined by Spearman rank correlation analysis (Table 2). BM CTRP3 concentration was positively correlated with leptin concentrations (*p* < 0.033, *R*^2^ = 0.352, *n* = 37). CTRP3 was not associated with BMI or any other measured bioactive factor in BM.

**Table 2:** Correlations of measured biological variables and CTRP3 concentration.

	*R*^2^	*p*
Glucose (mg/dL)	0.106	0.53
Ghrelin (pg/mL)	0.102	0.55
Insulin (pg/mL)	0.057	0.74
Leptin (pg/mL)	0.352*	0.03
Adiponectin (ng/mL)	−0.017	0.92
IL-6 (pg/mL)	−0.089	0.60
TNF-α (pg/mL)	0.104	0.54
BMI (kg/m^2^)	0.284	0.09

**Notes:**

Correlations between measured variables and CTRP3 were analyzed by Spearman nonparametric correlation test.

* Denotes significant association.

## Discussion

The major findings of this study are that the novel adipokine CTRP3 is not only present in BM but also CTRP3 is significantly elevated in the BM of obese mothers (BMI > 30 kg/m^2^). CTRP3 is an exciting member of the CTRP family, which has been shown to have anti-diabetic and anti-inflammatory properties (Lin et al., 2017; Petersen et al., 2016; Peterson, 2016; Peterson, Wei & Wong, 2010; Yoo et al., 2013; Zhang et al., 2017). This may be particularly physiologically relevant as infant intestinal tight junctions remain open during this early neonatal development (Shehadeh, Sukhotnik & Shamir, 2006) and leptin has been demonstrated to be absorbed through the intestinal barrier in neonatal rat pups (Sanchez et al., 2005). Due to the anticipated protective properties of CTRP3, we initially anticipated that CTRP3 levels would be decreased in the BM from obese mothers. However, BM CTRP3 levels were elevated with obesity, which was contrary to our initial hypothesis. Nevertheless, this finding is in line with a recent paper showing that CTRP3 levels increase with obesity in females (Wagner et al., 2016). CTRP3, combined with leptin and other bioactive components, may be acting to prevent the maternal transfer of obesity, through reducing food intake, stimulating metabolism, or a yet to be determined mechanism. On the other hand, elevated levels of CTRP3 may negatively influence infant development, as CTRP3’s function during growth and development is unknown.

We found that CTRP3 levels are highly variable in both normal weight and obese mothers with concentrations of 2–80 ng/mL. This high variability in BM CTRP3 levels indicate that a number of factors beyond pregravid BMI contribute to CTRP3’s regulation. In addition, leptin, which was positively correlated with CTRP3, is not only produced by adipose tissue, but also is expressed directly from the mammary gland (Smith-Kirwin et al., 1998), indicating that the mammary gland may also express and regulate CTRP3 concentration in BM. However, as CTRP3 levels are upto 10 times higher in human circulation (200–1,000 ng/mL) (Wagner et al., 2016), CTRP3 may be absorbed from the circulation and not necessarily be actively synthesized by the mammary gland. Therefore, to determine the true source of BM CTRP3, future studies should examine mammary gland CTRP3 expression, the association of circulating and BM CTRP3 levels, and the transfer of a labeled recombinant CTRP3 from the circulating to the milk.

Further, findings from this study independently confirm previous work that BM from obese mothers has higher concentration of insulin and leptin, with no changes observed between the two populations in ghrelin, IL-6, TNF-α, or glucose levels (Fields, Schneider & Pavela, 2016; Kugananthan et al., 2017; Lemas et al., 2016; Socha et al., 2016; Stern, Rutkowski & Scherer, 2016; Young et al., 2017). Although some studies have reported an association between BMI and BM adiponectin levels, most studies do not find a relationship (Andreas et al., 2014; Fields, Schneider & Pavela, 2016; Kugananthan et al., 2017; Ley et al., 2012). The data from this study also found no correlation with maternal obesity and BM adiponectin levels.

Cross-sectional evidence suggests that neonatal development is a crucial time-period for preventing obesity and metabolic dysfunction throughout life (Çatli, Olgaç Dündar & Dündar, 2014; Zalewski et al., 2017). Further, experimental evidence in animal models (offspring of normal weight dams that were suckled by obese dams) demonstrated an exaggerated metabolic phenotype in adulthood (Oben et al., 2010). However, the mechanisms that are responsible for creating the predisposition to obesity have not been identified. BM is a complex solution that contains a number of bioactive factors that have the potential to significantly affect appetite, growth, development, and overall health (Çatli, Olgaç Dündar & Dündar, 2014; Fields, Schneider & Pavela, 2016; Savino et al., 2002). For example, circulating leptin levels are higher in breastfed compared to formula fed children (Savino et al., 2002) and studies indicate that leptin transfers from the milk to the infant’s circulatory system (Casabiell et al., 1997; Savino et al., 2002). These data support the hypothesis that non-nutritive factors such as leptin have the potential to influence neonatal development.

## Conclusion

The results from this study support the hypothesis that maternal adiposity changes hormonal and immunological components of BM.

### Study limitations

The primary limitations of this study are that we were unable to examine the association of CTRP3 in the BM with circulatory levels in the mothers or the infants. These data would allow the investigators to determine if maternal circulating levels of the various factors would predict the concentration in the BM and more importantly, the amount and concentration of CTRP3 provided to the infants. Additionally, we were unable to examine relationships between CTRP3 levels in the BM and current maternal or infant anthropometric data or other markers of infant health. Future studies will need to determine the role CTRP3 and other novel secreted humoral factors have on infant development. Further, due to the preliminary nature of this study, we only acquired a single BM sample from each mother, future studies will need to determine if BM CTRP3 levels change throughout the postnatal period. Lastly, the cross-sectional nature of this study precludes making causal claims.

## Supplemental Information

10.7717/peerj.4472/supp-1 Supplemental Information 1 Values for each subject. The values for bioactive components measured in the collected breast milk for each subject are reported. Additional data include subject height, weight, age, and the number of days postpartum day that the sample was collected.

10.7717/peerj.4472/supp-2 Supplemental Information 2 Associations of measured bio-active components. Unbiased associations between measured variables were analyzed by Spearman nonparametric correlation test.

10.7717/peerj.4472/supp-3 Supplemental Information 3 Full immunoblot image for Fig. 1A. Chemiluminescence image overlaid with bright-field image so that ladder is visable (lane 0). Human Milk Insulin is Related to Maternal Plasma Insulin and BMI–But other Components of Human Milk do not Differ by BMI Immunoblot analysis of whole breast milk (lane 1), unbound protein solution samples, (lanes 2–5) and immunoprecipitate samples (lanes 6–9). BM samples were immunoprecipitated with vehicle only (lanes 2 & 6), normal serum (lanes 3 & 7), anti-CTRP1 (lanes 4 and 8), or anti-CTRP3 antibody (lanes 5 and 9).

## Additional Information and Declarations

### Data Availability

The following information was supplied regarding data availability: The raw data has been supplied as Supplemental Dataset Files.
