# Supplementary figures and images for: The adipokine C1q TNF related protein 3 (CTRP3) is elevated in the breast milk of obese mothers
Source: PeerJ. 2018 Mar 5;6:e4472. doi: 10.7717/peerj.4472 (PMC5842766; doi:10.7717/peerj.4472)

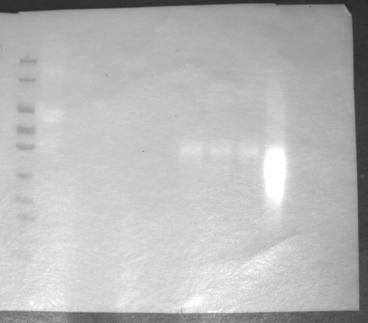

Supplement: Supplemental Information 3 — Chemiluminescence image overlaid with bright-field image so that ladder is visable (lane 0). Human Milk Insulin is Related to Maternal Plasma Insulin and BMI–But other Components of Human Milk do not Differ by BMI Immunoblot analysis of whole breast milk (lane 1), unbound protein solution samples, (lanes 2–5) and immunoprecipitate samples (lanes 6–9). BM samples were immunoprecipitated with vehicle only (lanes 2 & 6), normal serum (lanes 3 & 7), anti-CTRP1 (lanes 4 and 8), or anti-CTRP3 antibody (lanes 5 and 9). [file peerj-06-4472-s003.png]
